# Tiaoshen Tongluo Attenuates Fibrosis by Modulating the TGF-*β*1/Smad Pathway in Endometrial Stromal Cells and a Rat Model of Intrauterine Adhesion

**DOI:** 10.1155/2021/6675329

**Published:** 2021-04-23

**Authors:** Hongping Niu, Xiaoling Miao, Xingxiu Zhan, Xiaona Zhou, Xingyan Li, Lijuan Jiang

**Affiliations:** ^1^Department of Gynecology, The First Affiliated Hospital of Nanjing University of Chinese Medicine, Nanjing, Jiangsu, China; ^2^Department of Gynecology, The First Affiliated Hospital of Yunnan University of Chinese Medicine, Kunming, Yunnan, China; ^3^Office of Graduate Management, The First Affiliated Hospital of Yunnan University of Chinese Medicine, Kunming, Yunnan, China; ^4^The Traditional Chinese Medicine Health Service Center, The First Affiliated Hospital of Yunnan University of Chinese Medicine, Kunming, Yunnan, China

## Abstract

Intrauterine adhesion (IUA) is a serious complication caused by excessive fibrosis resulting from endometrial repair after trauma. The traditional Chinese medicine Tiaoshen Tongluo recipe (TTR) contains ingredients associated with the alleviation of fibrosis. The transforming growth factor-*β*1 (TGF-*β*1)/Smad pathway is thought to mediate fibrosis in IUA. In this study, we evaluated the influence of TTR on endometrial fibrosis in a rat model of IUA and in TGF-*β*1-stimulated endometrial stromal cells (ESCs). TTR was found to alleviate the level of endometrial fibrosis in a rat model of IUA. A higher number of collagen fibers and greater damage were observed in the endometrial tissue of untreated rats compared to those treated with TTR. The expression of TGF-*β*1, Smad2, Smad3, and Smad4 was upregulated following IUA, whereas Smad7 expression was downregulated. TTR lowers the expression of TGF-*β*1, Smad2, Smad3, and Smad4 but increases the expression of Smad7 in vivo, indicating that TTR can modulate the expression of the TGF-*β*1/Smad pathway to mediate fibrosis. In ESCs, the phosphorylation of Smad2 and Smad3 and upregulation of Smad4 were induced by TGF-*β*1 whereas the expression of Smad7 was inhibited. Administration of TTR reduces the phosphorylation of Smad2 and Smad3, increases Smad4 expression induced by TGF-*β*1, and promotes the expression of Smad7. TTR modulates the TGF-*β*1/Smad pathway to alleviate the generation of fibrotic tissue in response to IUA.

## 1. Introduction

Intrauterine adhesion (IUA) can occur in patients with endometrial repair disorder following the uterine wall and endometrial trauma during pregnancy and is frequently associated with infertility and an increased risk of miscarriage [1, 2]. Therapeutic options are limited but include high doses of estrogen because hormonal changes in the uterus are thought to increase susceptibility to injury [3, 4]. Excessive endometrial fibrosis is associated with IUA and could be related to the failure of the normal wound healing process [5]. Therefore, the expression of endometrial stem cell markers and those associated with fibrosis are upregulated in endometrial tissue with IUA [6].

The multifunctional cytokine transforming growth factor-*β*1 (TGF-*β*1) plays a major role in the stimulation of extracellular matrix (ECM) proteins and inhibition of ECM degradation during the process of fibrosis in IUA [7]. In fact, the levels of TGF-*β*1 and the Smad pathway proteins it regulates are correlated with IUA [7]. Recently, stem cell-derived exosomes were found to reverse the elevated expression of TGF-*β*1 and Smad mRNA in endometrial epithelial cells and an animal model of IUA [8]. Moreover, the suppression of the TGF-*β*1/Smad pathway by drug intervention is proposed to inhibit endometrial fibrosis [9]. TGF-*β*1 is activated in the human endometrium by serine kinases; once activated, it, in turn, activates Smad2, Smad3, and Smad4 [10], which mediate most profibrotic activities [11]. Therefore, TGF-*β*1 can be used to induce IUA in endometrial stromal cells (ESCs) and provide a target for the alleviation of endometrial fibrosis.

Traditional Chinese medicine herbal formulae are characterized by a holistic concept and generations of development [12]. Several traditional herbal medicines used to treat fibrosis have been found to function by subduing the TGF-*β*1/Smad pathway [13–17]. The expression levels of TGF-*β*1 and Smad3 were decreased in a carbon tetrachloride-induced liver fibrosis model in rats treated with the herbal decoction JinSanE, and Smad7 expression was found to increase [13]. Similarly, the traditional Chinese medicine, hydroxysafflor yellow A (HSYA), significantly reduced carbon tetrachloride-induced liver fibrosis through decreased TGF-*β*1 expression and phosphorylation of Smad4 [17]. The extract of Xin Jia Xuan Bai Cheng Qi decoction was found to alleviate a bleomycin-induced model of pulmonary fibrosis in rats by inhibiting TGF-*β*1 and Smad2 expression, while increasing the activation of Smad7 [14]. Likewise, Qishen granules were found to attenuate cardiac fibrosis by inhibiting the TGF-*β*/Smad3 pathway [15].

In the present study, we assessed whether a Tiaoshen Tongluo recipe (TTR) could prevent fibrosis by modulating the TGF-*β*1/Smad pathway. TTR contains ingredients that are associated with reproductive health (e.g., Semen Cuscuta) [18], pain relief (e.g., *Achyranthes bidentata*) [19], inflammatory properties (e.g., *Angelica sinensis*) [20], and antioxidant effects (e.g., Radix Astragali) [21]. Some of the ingredients in TTR are known to influence the levels of TGF-*β*1 [22]. In particular, *Angelica sinensis* has been found to reduce fibrosis and prevent epidural scar adhesion in a postlaminectomy rat model by inhibiting the expression of TGF-*β*1 [23]. In this study, the antifibrotic effects of TTR were investigated in TGF-*β*1-stimulated ESCs and a rat model of IUA.

## 2. Materials and Methods

### 2.1. Chinese Herbal Medicine

Dispensing granules of TTR were provided by the Yunnan Provincial Hospital of Traditional Chinese Medicine. The TTR dispensing granules were composed of Semen Cuscuta, 30 g; cooked Rehmannia, 15 g; tuckahoe, 15 g; Rhizoma Polygonati, 30 g; Fructus Aurantii, 15 g; *Achyranthes bidentata*, 10 g; *Angelica sinensis*, 15 g; *Ligusticum wallichii*, 15 g; Alisma, 15 g; Jiang Magnolia, 15 g; peach kernel, 10 g; red Paeonia, 15 g; *Atractylodes*, 15 g; Radix Astragali preparata, 30 g; Lindera aggregate, 15 g; and Vinegar rhizoma zedoariae, 15 g. Before each gavage, the TTR granules were diluted to 2 g/ml with boiling water.

### 2.2. Animals

Specific pathogen-free grade Sprague Dawley rats (female, approximately 250∼280 g) were purchased from the Shanghai Sip-Bikai Laboratory Animal Co. Ltd., Shanghai, China, and were housed five to a cage in 12 h light/dark cycles at room temperature and 45–55% humidity with food and water ad libitum. All animals were treated in accordance with the guidelines of the Ethics Committee of the Yunnan University of Chinese Medicine.

### 2.3. IUA Model

A dual-injury method was used to create the IUA model in rats as described previously [24]. At 10 weeks, 20 rats with regular 4-5 day estrous cycles were anesthetized with an intraperitoneal injection of 1% pentobarbital sodium and placed in a supine position. A 2 cm transverse lower abdominal incision was made down to the peritoneal cavity to expose the uterine horns. The right uterine horn was used for all procedures. The left uterine horn was used as the control. A mechanical injury was created by rotating a 16-gauge needle inside the fallopian tube through a small incision. When bleeding occurred, the uterine horn was flushed with saline and then sutured. After a mechanical injury, a 5 cm length of cotton thread soaked in lipopolysaccharide (0.6 mg/L) was inserted into the uterus and left for 48 h. The incisions were sutured and the animals were allowed to recover. After the IUA operation, the rats were divided into four groups and given 0, 2.875, 5.75, and 11.5 g/kg TTR by gavage over 8 weeks. The rats were then sacrificed and the uteri were removed.

### 2.4. Histological Analysis

For hematoxylin and eosin (H&E) and Masson staining, the uteri were collected, fixed with 4% concentration of paraformaldehyde overnight, dehydrated in stratified alcohol, and then embedded in paraffin. Sections (5 *μ*m) were stained with H&E and Masson trichrome (Solarbio, Beijing, China) according to the manufacturer's protocols. More than five uterine cross sections were assessed from each group. Endometrial thickness was measured with ImageJ software and the level of fibrosis was estimated using a scale of 0–3, with 3 being the most intense blue staining and the highest level of fibrosis. Capillary vessels were counted under 100x magnification.

### 2.5. Immunohistochemistry

Immunohistochemistry was performed on dewaxed and hydrated paraffin-embedded 5 *μ*m sections of endometrial tissue using antibodies against vimentin (1 : 200, Abcam, Cambridge, UK) and Smad4 (1 : 50, Santa Cruz Biotechnology, Dallas, TX, USA). Specimens were blocked in 1% bovine serum albumin (BSA) for 15 mins and incubated with primary antibody overnight at 4°C. They were then incubated with a secondary antibody for an hour at room temperature and observed under a fluorescence microscope (Nikon, Tokyo, Japan).

### 2.6. Drug-Containing Serum Preparation

The rats were divided into a drug serum group, given TTR granules (11.5 g/kg) by gavage, and a normal serum group, given the same volume of saline twice a day for 3 consecutive days. The rats fasted for 12 hours after and were given TTR granules for 1 day. Blood was collected from the heart under sterile conditions 1 hour later and left to stand for 2 h. The supernatant was centrifuged for 10 min in a high-speed centrifuge, and the resulting serum was treated as drug-containing serum.

### 2.7. Endometrial Stromal Cell (ESC) Isolation

Because IUA is usually caused by endometrial trauma during pregnancy, we isolated ESCs from rats during early pregnancy for an *in vitro* study. Uteri were collected from sacrificed rats on day 4 of pregnancy. The uteri were minced and incubated in Dulbecco's modified Eagle medium (DMEM) with 1 mg/ml collagenase for 1 h at 37°C with shaking (110 rpm). Cell debris was allowed to settle and the supernatant was centrifuged. The endometrial epithelial and stromal cells in the resultant pellet were separated with a 40 *μ*m cell strainer. Decidualization was performed on stromal cells that passed through the strainer. After growing cells to 70% confluency in DMEM they were treated with 0.5 mM cAMP and 100 nM medroxyprogesterone acetate.

### 2.8. Immunodiagnosis of ESCs

ESCs were fixed in 4% paraformaldehyde on coverslips for 20 min and then permeabilized with 0.5% Triton X-100 for 10 min. The ESCs were blocked in 3% BSA, washed in PBS, and then incubated with anti-vimentin monoclonal antibodies (1 : 100, Abcam) overnight at 4°C. They were then incubated with a secondary antibody for an hour at room temperature. Cells were counterstained with DAPI (Beyotime, Shanghai, China) and observed under a fluorescence microscope (Nikon).

### 2.9. Cell Treatment

To induce fibrotic characteristics, the ESCs were treated with 0, 10, 20, 50, and 100 ng/ml TGF-*β*1 for 48 h. To assess the effects of TTR, the ESCs were first treated with 50 ng/ml TGF-*β*1 for 48 h. Then, normal rat serum or serum containing different volumes of TTR (5%, 10%, or 20%) was added. The cells were cultured for a further 72 hours.

### 2.10. Real-Time PCR

Total RNA was extracted from ESCs and endometrial tissues using Trizol reagent (Invitrogen, Carlsbad, CA, USA). After measuring the quality and quantity of the RNA with a NanoDrop ND 1000 instrument (NanoDrop Technologies, Wilmington, DE, USA), the RNA was reverse transcribed using M-MLV reverse transcriptase and RNase inhibitor (Promega, Madison, WI, USA). The RNA expression of GAPDH (control), Smad2, Smad3, Smad4, and Smad7 was determined using the primers specified in Table 1 and amplified with SYB Green PCR Master Mix (Applied Biosystems, Foster City, CA, USA) following the manufacturer's instructions. Expression levels of mRNA were determined using the 2^−ΔΔCt^ method and normalized to the control.

### 2.11. Western Blot Analysis

ESCs and endometrial tissues were first lysed with lysis buffer and centrifuged at 12,000 × *g* for 15 min at 4°C. A BCA protein assay kit (Beyotime) was used to determine the quantity of protein. Protein samples (50 *μ*g) were separated with SDS-PAGE and transferred to polyvinylidene fluoride (PVDF) membranes. The PVDF membranes were blocked with 5% nonfat milk in TBST (10 mM Tris-HCl, 100 mM NaCl, 0.1% Tween-20, pH 7.4) for an hour at room temperature and then incubated in primary antibody overnight at 4°C. The primary antibodies used in this study were rabbit anti-*β*-actin (1 : 2500, control), anti-TGF-b1 (1 : 1000), anti-p-Smad2 (1 : 500), anti-Smad2 (1 : 1000), anti-p-Smad3 (1 : 1000), anti-Smad3 (1 : 500), anti-Smad4 (1 : 200), and anti-Smad7 (1 : 500) all from Santa Cruz Biotechnology. Membranes were then incubated with horseradish peroxidase-labeled goat anti-rabbit secondary antibody (1 : 10,000) for an hour at room temperature. Protein bands were visualized using Luminata Crescendo Western HRP Substrate (Millipore, Billerica, MA, USA) and a molecular imager (Bio-Rad, Philadelphia, PA, USA). Densitometry analysis was determined relative to *β*-actin using 1-D Analysis Software (National Institutes of Health, USA).

### 2.12. Statistical Analysis

All data are expressed as the means ± standard deviation from three independent experiments. One-way analysis of variance and Dunnett's post hoc multiple comparison were used to determine statistical significance. Data were analyzed using GraphPad Prism 5 (GraphPad, CA, USA). A value of *P* < 0.05 was considered significant.

## 3. Results

### 3.1. TTR Alleviates Endometrial Fibrosis in a Rat Model of IUA

**Figure 1:**
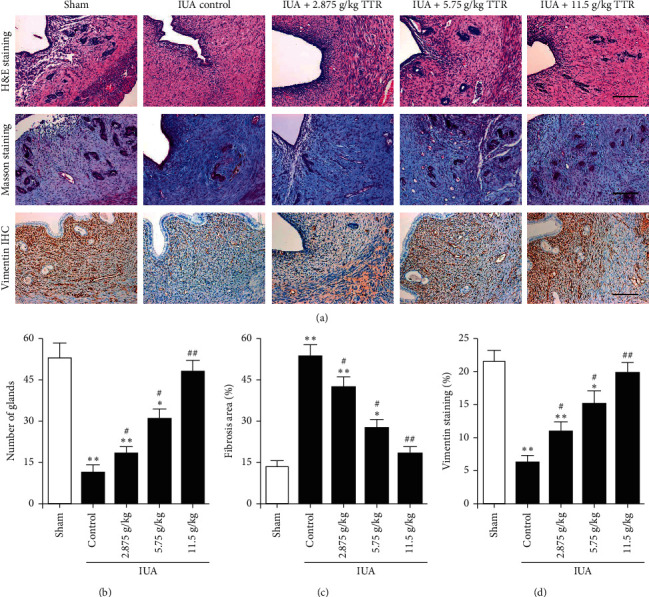
Tiaoshen Tongluo recipe (TTR) alleviates the fibrosis caused by intrauterine adhesion (IUA) in rat endometrium. Following the IUA procedure, the rats were given either 2.875 g/kg, 5.75 g/kg, or 11.5 g/kg TTR by gavage for 8 weeks. Rat endometrium was collected from each group. (a) Representative images of hematoxylin and eosin (H&E), Masson, and vimentin immunohistochemistry (IHC) staining in rat endometrium sections. Scale bar = 100 *μ*m. (b–d) Statistical results of gland number (b), changes in intrauterine fibrosis (c), and the percent of vimentin-positive areas (d) after endometrial damage and treatment. ^*∗*^*P* < 0.05, ^*∗∗*^*P* < 0.01 compared to sham group, ^#^*P* < 0.05, ^##^*P* < 0.01 compared to IUA control group.

We first assessed whether TTR could alleviate IUA in a rat model by examining the level of fibrosis in endometrial tissue (Figure 1(a)). The number of endometrial glands was significantly reduced in the IUA model compared with the sham-operated rats (*P* < 0.01, Figure 1(b)). However, the number of glands increased dose-dependently in rats that were given TTR. We used Masson's trichrome staining to detect collagen fibers in the endometrial tissue. Endometrial fibrosis increased significantly in the IUA model but TTR was able to decrease the area of fibrosis dose-dependently until levels nearly reached those of the sham-operated rats (Figure 1(c)). In the IUA control group, there were a higher number of collagen fibers and more damage was observed in the endometrial tissue than in the IUA TTR-treated groups. To determine the regeneration of endometrial cells following the IUA procedure, we stained the endometrial tissue with vimentin (Figure 1(d)). A higher level of staining was observed in the sham-operated rats and in those treated with high doses of TTR, indicating that TTR could promote stromal and endothelial cell regeneration after IUA. Overall, our histological results demonstrated that IUA increased the level of fibrosis in endometrial tissue whereas treatment with TTR could alleviate this damage.

### 3.2. TGF-*β*1/Smad Pathway Is Moderated by TTR in IUA

**Figure 2:**
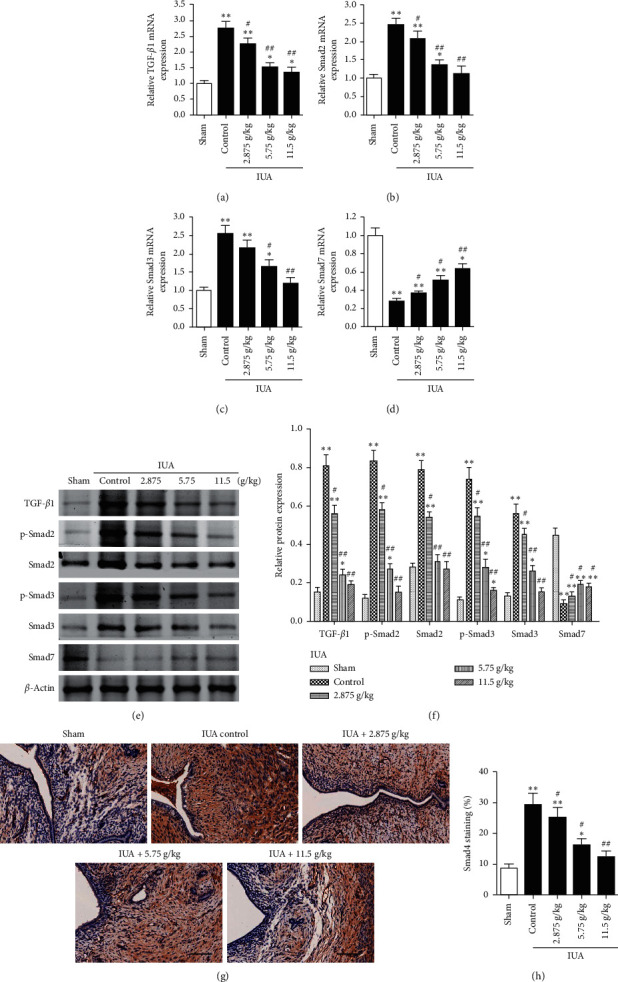
Influence of the Tiaoshen Tongluo recipe (TTR) on the TGF-*β*1/Smad pathway in a rat model of intrauterine adhesion (IUA). The relative mRNA expression of TGF-*β*1 (a), Smad2 (b), Smad3 (c), and Smad7 (d) detected by real-time PCR in rat endometrium. (e) The protein expression of TGF-*β*1, Smad2, phosphorylated-Smad2, Smad3, phosphorylated-Smad3, and Smad7 detected by Western blot. (f) The protein expression of TGF-*β*1, Smad2, phosphorylated-Smad2, Smad3, phosphorylated-Smad3, and Smad7 was quantified relative to that of *β*-actin. (g) Smad4 was detected by IHC staining in rat endometrium of each group. Scale bar = 100 *μ*m. (h) The percent of Smad4-positive areas was quantified. ^*∗*^*P* < 0.05, ^*∗∗*^*P* < 0.01 compared to the sham group; ^#^*P* < 0.05, ^##^*P* < 0.01 compared to the IUA control group.

To establish whether TTR influenced proteins that are upregulated in fibrosis, we next examined the activity of the TGF-*β*1/Smad pathway in the rat endometrium following IUA and in rats treated with various doses of TTR (2.88, 5.75, and 11.50 g/kg). Real-time PCR indicated that the relative expression of TGF-*β*1, Smad2, and Smad3 was upregulated in the IUA model, whereas the expression of Smad7 was downregulated (Figures 2(a)–2(d)). However, after TTR treatment, the levels of TGF-*β*1, Smad2, and Smad3 were significantly lower than in the untreated IUA model and at the highest dose of TTR, there was no significant difference in the expression of Smad2 and Smad3 in the IUA model compared with the sham-operated rats (Figures 2(a)–2(c)). In contrast, the level of Smad7 expression increased dose-dependently with TTR; the highest level of Smad7 expression was obtained at the highest dose of TTR (Figure 2(d)).

Similar results were obtained in the analysis of protein levels and phosphorylation by western blotting and immunohistochemistry (Figures 2(e)–2(h)). Levels of TGF-*β*1, phosphorylated Smad2 and Smad3 (Figures 2(e) and 2(f)), and Smad4 (Figures 2(g) and 2(h)) were the highest in the untreated IUA model. Following TTR, the levels decreased. In contrast, the level of Smad7 was the highest in the sham-operated rats with lower levels found after IUA (Figures 2(d)–2(f)). These results indicate that the TGF-*β*1/Smad pathway is activated following IUA. TTR seems to inhibit the upregulation of the TGF-*β*1/Smad pathway following injury and reduces the fibrotic response in the rat endometrium.

### 3.3. Activation of the Smad Pathway by TGF-*β*1 In Vitro

**Figure 3:**
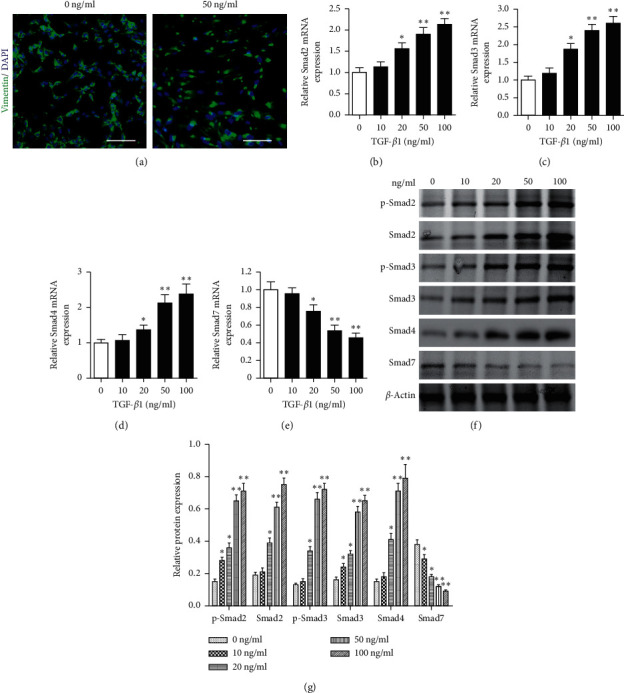
Activation of Smad proteins by TGF-*β*1 in vitro. (a) Representative immunofluorescence-based identification of endometrial stromal cells (ESCs) with or without 50 ng/ml TGF-*β*1 treatment for 48 h. Images showing vimentin staining (green). Nuclei were counterstained with DAPI (blue). Scale bar = 100 *μ*m. (b–g) The ESCs were treated with 0, 10, 20, 50, and 100 ng/ml TGF-*β*1 for 48 h. The relative mRNA expression of Smad2 (b), Smad3 (c), Smad4 (d), and Smad7 (e) detected by real-time PCR. (f) The protein expression of Smad2, phosphorylated-Smad2, Smad3, phosphorylated-Smad3, Smad4, and Smad7 detected by Western blot. (g) The protein expression of Smad2, phosphorylated-Smad2, Smad3, phosphorylated-Smad3, Smad4, and Smad7 was quantified relative to that of *β*-actin. ^*∗*^*P* < 0.05, ^*∗∗*^*P* < 0.01 compared to control without TGF-*β*1 (0 ng/ml).

To determine the involvement of TGF-*β*1 in the activation of the Smad pathway, we examined the activation of the Smad pathway in ESCs with TGF-*β*1 applied exogenously. As shown in Figure 3(a), TGF-*β*1-stimulated ESCs had higher levels of vimentin expression than untreated ESCs, demonstrating that TGF-*β*1 was promoting fibrosis. The expression of Smad2, Smad3, Smad4, and Smad7 in ESCs in response to exogenous TGF-*β*1 was quantified by real-time PCR (Figures 3(b)–3(e)). With the application of increasing concentrations of TGF-*β*1, Smad2, Smad3, and Smad4 mRNA expression increased in a dose-dependent manner, whereas the levels of Smad7 decreased. No significant differences were recorded at TGF-*β*1 levels of 10 ng/ml. However, the highest expression of Smad2, Smad3, and Smad4 and the lowest expression of Smad7 were recorded with TGF-*β*1 levels of 100 ng/ml. Western blotting revealed similar findings (Figures 3(f) and 3(g)). The phosphorylation of Smad2 and Smad3 and expression levels of Smad4 increased with an increasing concentration of TGF-*β*1, whereas Smad7 levels decreased. These findings indicate that TGF-*β*1 can modulate the expression of the Smad pathway in vitro.

### 3.4. Relative Smad Activity in TGF-*β*1-Stimulated ESCs following Treatment with TTR

**Figure 4:**
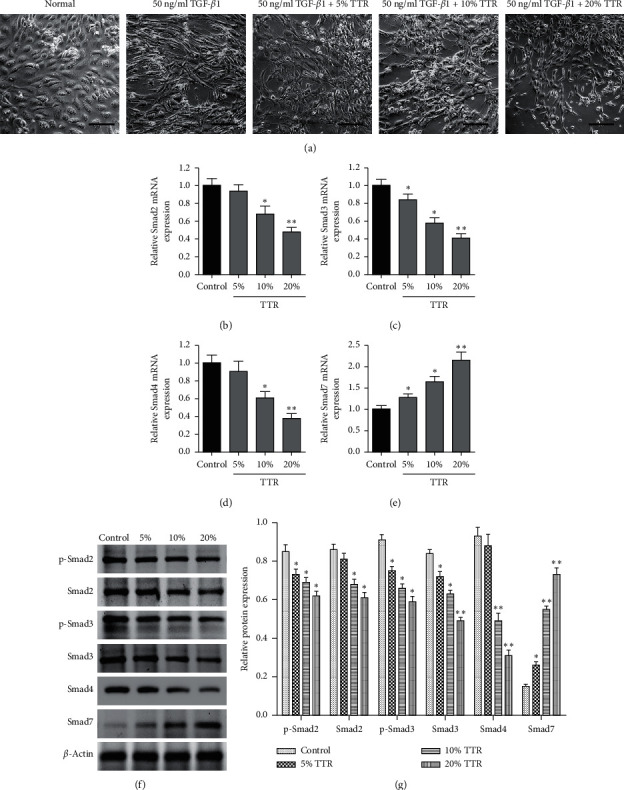
Relative Smad activity following treatment with the Tiaoshen Tongluo recipe (TTR). After treating ESCs with 50 ng/ml TGF-*β*1 for 48 h, normal rat serum and drug-containing serum of different volumes were added. ESCs treated with TTR-containing serum at final concentrations of 5%, 10%, or 20% were cultured for a further 72 hours. The blank control group was treated with 15% normal rat serum. (a) Representative images showing the morphology of ESCs after different treatments. Scale bar = 100 *μ*m. The relative mRNA expression of Smad2 (b), Smad3 (c), Smad4 (d), and Smad7 (e) detected by real-time PCR. (f) The protein expression of Smad2, phosphorylated-Smad2, Smad3, phosphorylated-Smad3, Smad4, and Smad7 assessed by Western blotting. (g) The protein expression of Smad2, phosphorylated-Smad2, Smad3, phosphorylated-Smad3, Smad4, and Smad7 was quantified relative to that of *β*-actin. ^*∗*^*P* < 0.05, ^*∗∗*^*P* < 0.01 compared to control.

We next determined whether TTR would have an effect on ESCs treated with 50 ng/ml TGF-*β*1 for 48 h. Rat serum containing TTR was added to the ESCs at concentrations of 5%, 10%, and 20% and then cultured for further 72 hours. ESC morphology was observed by microscopy (Figure 4(a)). Cells that were not stimulated by TGF-*β*1 were round or oval, with a full cytoplasm, and were arranged in dense rows like paving stones, whereas treatment with 50 ng/ml TGF-*β*1 for 48 h led to a decrease in cell density. Furthermore, most of the TGF-*β*1-induced cells were longer and thinner in shape than unstimulated cells. The addition of TTR inhibited the morphological changes induced by TGF in a concentration-dependent manner (Figure 4(a)). Levels of Smad2 (Figure 4(b)), Smad3 (Figure 4(c)), Smad4 (Figure 4(d)), and Smad7 (Figure 4(e)) mRNA expression were then detected by real-time PCR. The addition of TTR significantly lowered the expression of Smad2, Smad3, and Smad4 in TGF-*β*1-stimulated ESCs (Figures 4(b)–4(d)), whereas Smad7 expression was increased (Figure 4(e)). Western blotting indicated that the addition of TTR decreased the phosphorylation of Smad2 and Smad3, as well as Smad4 expression in TGF-*β*1-stimulated ESCs, and increased Smad7 expression (Figures 4(f) and 4(g)).

Overall, these results indicate that TTR can modulate the TGF-*β*1/Smad pathway to moderate the generation of fibrotic tissue in response to the injury repair process in endometrial tissue.

## 4. Discussion

IUA is considered to be a fibrotic disease in endometrial tissue [25]. Fibrosis causes endometrial cells to lose their sensitivity toward hormones, which leads to long-term complications [26]. The exact mechanism is not fully understood but is thought to involve pathways central to ECM formation such as the TGF-1*β*/Smad signaling pathway [6, 7, 27–29]. The therapeutic options for IUA are limited. However, several herbal medicines have been used to attenuate the generation of fibrotic tissue [30], especially in relation to hepatic [31], pulmonary [32], and renal [33] fibrosis.

In this study, we evaluated the use of the herbal medicine TTR in alleviating fibrosis associated with IUA. We found that TTR reduced the level of fibrosis in endometrial tissue in a double-injury model of IUA in rats. IUA increased the expression level of fibrosis-associated genes including TGF-*β*1, Smad2, Smad3, and Smad4. The TGF-*β*1 signaling pathway has been implicated in the pathobiology of fibrosis [34]. Activation of TGF-*β*1 leads to phosphorylation of Smad2 and Smad3, which then form a complex with Smad4. This Smad2/3/4 complex then translocates to the nucleus, where it regulates the transcription of specific genes [35]. Here, IUA-induced increases in Smad4 and TGF-*β*1 expression, and Smad2 and Smad3 phosphorylation were reduced after treatment with TTR. Similar changes in Smad2, Smad3, and Smad4 have been observed in previous studies examining potential antifibrotic compounds on several types of fibrosis [36–40]. For example, in liver fibrosis, the antifibrotic effects of propylene glycol alginate sodium sulfate involved the suppression of TGF-*β*1, Smad2, and Smad3 [36]. In a rat model of unilateral ureteral obstruction (UUO), oleanolic acid was found to decrease the expression of TGF-*β*1, the phosphorylation of Smad2, and the mRNA expression of collagen I, collagen III, and fibronectin [37]. The Traditional Chinese Medicine compound HuangQi was found to improve kidney fibrosis in UUO mice by downregulating TGF-*β*1, Smad4, Smad2/3, and phosphorylated-Smad2/3, while upregulating Smad7 [41]. Hoi et al. [39] found that there was crosstalk between WNT/*β*-catenin and TGF-*β*/SMAD signaling and that a WNT/*β*-catenin inhibitor could improve TGF-*β*1-induced renal fibrosis. In pulmonary fibrosis, nagilactone D, a dinorditerpenoid derived from *Podocarpus nagi*, attenuated the TGF-*β*1-induced expression of collagen I, collagen III, and fibronectin in human pulmonary fibroblasts and suppressed the phosphorylation of Smad2 [40].

In our study, we found that Smad7 is downregulated when the phosphorylation of Smad2/3 and expression levels of Smad4 are increased. This result replicates similar results found in other studies [42–44]. A number of these studies have implicated microRNAs in the regulation of Smad7. Tao et al. [44] found that Smad7 was one of the target genes of miR-216a. The reduced expression of Smad7 through the interaction with miR-216a led to the activation of TGF-*β*1 and the consequent phosphorylation of Smad2, leading to increased fibrotic characteristics in human cardiac fibroblasts. miR-21, in particular, is often associated with the suppression of Smad7 and the subsequent induction of TGF-*β*1 [43, 45, 46]. Corilagin, a plant-derived ellagitannin, was found to modulate fibrosis through the miR-21/Smad7/ERK signaling pathway in a mouse model of hepatic fibrosis [47]. Therefore, mounting evidence suggests that Smad7 is a negative regulator of the TGF-*β*/SMAD pathway and prevents TGF-*β*1-mediated fibrosis [48]. We found that the induction of Smad2, Smad3, and Smad4 by elevated levels of TGF-*β*1 increased fibrotic characteristics in a rat model of IUA and that levels of Smad7 are reduced. However, our results suggest that increasing levels of TGF-*β*1 could suppress the level of Smad7 in ESCs. The administration of TTR reduces the levels of Smad2, Smad3, and Smad4 and upregulates the level of Smad7, but this could be through the suppression of TGF-*β*. In our study, although the exact mechanism of regulation is unclear, the high expression of Smad7 signifies a reduction in fibrogenesis.

## 5. Conclusion

In the present study, we have demonstrated that TTR modulates fibrosis in a rat model of IUA. We have shown that TTR upregulates Smad7 and downregulates TGF-*β*1, which prevents the phosphorylation of Smad2/3 and expression of Smad4, and the subsequent regulation of genes involved in fibrogenesis. We propose that TTR may assist in the prevention of IUA and could be considered as a therapeutic option to moderate the generation of fibrotic tissue in endometrial tissue.

## Figures and Tables

**Table 1:** Primers used in the study.

	Primer sequence 5′⟶3′
GAPDH-F	TGCTGGTGCTGAGTATGTCG
GAPDH-R	TCATGAGCCCTTCCACGATG
Smad2-F	GAGACACCAGTCTTGCCTCC
Smad2-R	CGGAGAGCCTGTGTCCATAC
Smad3-F	TTCCATCCCCGAGAACACTAAC
Smad3-R	GTGACTGGCTGTAGGTCCAAG
Smad4-F	TCCTGTGGCTTCCACAAGTC
Smad4-R	CAGGATGGGGCGGCATAG
Smad7-F	CTCCTGCTGTGCAAAGTGTTC
Smad7-R	ACAGTCTGCAGTTGGTTTGA

## Data Availability

The data are available from the corresponding author upon request.
